# A novel method for approximating equilibrium single-channel Ca^2+ ^domains

**DOI:** 10.1186/1471-2202-16-S1-P162

**Published:** 2015-12-18

**Authors:** Victor Matveev

**Affiliations:** 1 New Jersey Institute of Technology, NJ 07030, USA

## 

Localized calcium (Ca^2+^) signals control some of the most fundamental physiological processes, including synaptic transmission as well as its activity-dependent plasticity. Computational and mathematical modeling played a crucial role in the understanding of spatio-temporal Ca^2+ ^dynamics that drives these processes, and showed that Ca^2+ ^concentration around a single Ca^2+ ^channel reaches a quasi-stationary distribution (known as the Ca^2+ ^"nanodomain") within tens of microseconds after the opening of the channel, and collapses as rapidly after the closing of the channel. Such localization of Ca^2+ ^in time and space is achieved by its rapid diffusion as well as its binding to its multiple interaction partners collectively called Ca^2+ ^buffers and Ca^2+ ^sensors. One of the successes of mathematical modeling was the development of several analytic approximations describing the equilibrium concentration of Ca^2+ ^as a function of distance from the open Ca^2+ ^channel, such as the Rapid Buffering Approximation (RBA), the Linear Approximation (LA) and the Excess Buffering Approximation (EBA) [1-4]. Each of these approximations has a particular applicability parameter regime created by the interplay between the properties of Ca^2+ ^buffers, in particular their mobility and Ca^2+ ^binding rates, and the strength of the Ca^2+ ^current. Here we present a novel approximation method which does not rely on a specific range of the relevant Ca^2+ ^and buffer parameters, and is based on matching the low-distance and large-distance asymptotic behavior of the concentration function. Even at low orders, the resulting approximation is as accurate as the second-order RBA and EBA approximations [4], but its validity extends far beyond the parameter range of applicability of RBA and EBA. The usefulness of the resulting approximation is two-fold: first, together with the previously developed approximations, the novel method could provide a deeper intuition into the dependence of Ca^2+ ^nanodomain properties on the relevant buffering parameters, and second, it constitutes an efficient numerical approximation tool in the modeling of the Ca^2+ ^signals underlying presynaptic and postsynaptic phenomena.
